# Combined Knockout of RIPK3 and MLKL Reveals Unexpected Outcome in Tissue Injury and Inflammation

**DOI:** 10.3389/fcell.2019.00019

**Published:** 2019-02-20

**Authors:** Caroline Moerke, Florian Bleibaum, Ulrich Kunzendorf, Stefan Krautwald

**Affiliations:** Department of Nephrology and Hypertension, University Hospital Schleswig-Holstein, Kiel, Germany

**Keywords:** regulated cell death (RCD), necroptosis, *Ripk3/Mlkl*, ischemia- reperfusion injury, TNF-induced shock

## Abstract

Necroptosis, initially identified as a backup cell death program when apoptosis is hindered, is a prominent feature in the etiology and progression of many human diseases, such as ischemic injury and sepsis. Receptor-interacting protein kinase 3 (RIPK3) is the cardinal regulator of this cell death modality, recruiting and phosphorylating the executioner mixed lineage kinase domain-like protein (MLKL) to signal necroptosis, which is terminated by a cellular plasma membrane rupture and the leakage of intracellular contents from dying cells. Experimental data to date indicate that RIPK3 and MLKL is the core machinery essential for all necroptotic cell death responses. By using CRISPR/Cas9 (clustered regularly interspaced short palindromic repeat/CRISPR-associated protein 9) technology, we showed that *Ripk3* and *Mlkl* knockout and *Ripk3/Mlkl* double-knockout in necroptosis-sensitive cell lines extensively block susceptibility to necroptosis, in each case to an indistinguishable degree. *In vivo* studies using *Ripk3*- or *Mlkl*-deficient mice validated kidney ischemia reperfusion injury and high-dose tumor necrosis factor (TNF) availability, as druggable targets in necroptotic-mediated pathologies. Here, we demonstrated that *Ripk3* or *Mlkl*-deficient mice are protected to a similar extent from kidney ischemia reperfusion injury and TNF-induced toxicity. Remarkably, in contrast to each single knockout, *Ripk3/Mlkl* double-deficient mice did not have appreciable protection from either of the above necroptotic-mediated pathologies. Paradoxically, the double-knockout mice resembled, in each case, the vulnerable wild-type mice, revealing novel complexities in the mechanisms of inflammation-driven diseases, due to aberrant cell death.

## Introduction

Necroptosis is a caspase-independent programmed cell death mediated by receptor-interacting protein kinase 3 (RIPK3) activation (Cho et al., 2009; He et al., 2009; Zhang et al., 2009) and the pursuant RIPK3-mediated phosphorylation of its pseudokinase substrate mixed lineage kinase domain-like protein (MLKL) (Sun et al., 2012). This initial stimulus prompts a conformational change that results in MLKL oligomerization, plasma membrane translocation, and lethal permeation of the lipid bilayer, leading to the release of cellular content, which triggers an inflammatory response (Rickard et al., 2014b). However, the exact mechanism by which activated MLKL kills cells remains unclear (Petrie et al., 2017). The availability of pharmacological inhibitors, especially mice harboring deletions that are indispensable for necroptotic pathway signaling, facilitates research investigating the mechanisms of necroptosis and its relevance to diseases such as ischemic injury and sepsis (Krautwald et al., 2016). However, the role of natural necroptosis in human diseases remains controversial, and the potential off-target effects of the applied inhibitors besides kinase activity and existing scaffold functions of the involved proteins often complicate the interpretation of findings. In this context, our previously published data verify that pharmacologically blocking necroptosis may worsen diseases such as acute pancreatitis or vascular leakage syndrome, which is triggered by a high-dose tumor necrosis factor (TNF). The latter is often considered a model for systemic inflammatory response syndrome (SIRS) (Duprez et al., 2011). We recently discovered that necroptosis and ferroptosis, a caspase-independent regulated cell death modality characterized by the accumulation of lethal lipid reactive oxygen species (ROS) which is produced through iron-dependent lipid peroxidation, are alternative cell death pathways that operate in acute kidney failure, where each death modality can compensate for another when one is compromised (Müller et al., 2017). In contrast, we, along with others have reported that *Ripk3* deficiency and catalytically inactive RIPK1 are beneficial in renal ischemia-reperfusion injury (IRI), Gaucher’s disease, myocardial infarction, and the high-dose TNF shock model (Linkermann et al., 2013; Polykratis et al., 2014; Vitner et al., 2014; Zhang et al., 2016). Deleting either *Ripk3* or *Mlkl* can suppress skin inflammation in *RIPK1*-deficient mice (Dannappel et al., 2014), and the fact that *Ripk3* or *Mlkl* deficiency ameliorates liver inflammation and splenomegaly in *Sharpin*-deficient mice (Rickard et al., 2014a), suggesting that MLKL follows RIPK3 directly in necroptotic signaling, therefore confers a similar degree of protection against the abovementioned necroptotic-mediated injuries. Nevertheless, there are also studies indicating that *Mlkl* deficiency confers less protection in the kidney IRI model compared to *Ripk3* deficiency, and in contrast to *Ripk3*-deficient mice, *Mlkl*-deficient mice resemble wild-type mice in their sensitivity to hypothermia induced by low-dose TNF (Newton et al., 2016a). However, our present findings reveal that when mice experience severe renal IRI, or when treated intravenously with a high-dose TNF, the differences between *Ripk3*-deficient and *Mlkl*-deficient mice are less apparent, substantiating the premise that RIPK3 cannot exacerbate these injuries independently of MLKL. Interestingly, our findings describe for the first time that combined knockout of the necrosome members *Ripk3* and *Mlkl* in an entire organism antagonizes the beneficial effect of the respective single knockouts in necroptotic cell death processes of severe IRI and TNF-induced shock.

## Materials and Methods

### Cell Culture

NIH3T3 cells (American Type Culture Collection) were cultured in Dulbecco’s modified Eagle’s medium (Gibco/Thermo Fisher Scientific, Darmstadt, Germany) supplemented with 10% (vol/vol) fetal calf serum, 100 U/ml penicillin, and 100 μg/ml streptomycin in a humidified atmosphere containing 5% CO_2_. Generation of the CRISPR/Cas9 NIH3T3 knockout cells has been described previously (Müller et al., 2017). To exclude feasible off-target effects or clonal variations within the cell population, we generated and analyzed three guide RNAs per target gene and observed congruent outcomes in each case. Each gene knockout was validated via a western blot analysis of the protein expression, as described previously (Müller et al., 2017).

### Reagents and Antibodies

Recombinant purified TNFα, annexin V-fluorescein isothiocyanate (FITC) antibody, and 7-amino-actinomycin D (7-AAD) antibody was obtained from BioLegend (London, United Kingdom). The zVAD-fmk (herein referred to as zVAD) was obtained from Bachem (Weil, Germany); erastin and 1S,3R-RSL3 (herein referred to as RSL3) obtained from Tocris, Bio-Techne (Wiesbaden, Germany).

### Cell Death Detection *in vitro*

Phosphatidylserine exposure to the outer cell membrane of apoptotic cells, or at the inner plasma membrane of necrotic cells and 7-AAD incorporation into necrotic cells, was quantified by fluorescence-activated cell sorting (FACS). Staining was performed according to the manufacturer’s instructions (BioLegend). Fluorescence was analyzed using an FC 500 flow cytometer (Beckman Coulter, Krefeld, Germany).

### Mice

All mice (8 weeks old) used were on C57BL/6 background and age-, sex-, and weight-matched. The mice were received and independently bred as wild-type, *Ripk3* knockout, *Mlkl* knockout, and *Ripk3/Mlkl* double-knockout colonies, and mice of different genotypes were not housed in the same cages. The *Ripk3* and *Mlkl* single knockout mice as well as the *Ripk3/Mlkl* double-knockout (dko) mice have been described previously (Newton et al., 2004; Murphy et al., 2013; Tanzer et al., 2015). All mice were kept on a standard diet and a 12-h day/night rhythm. All *in vivo* experiments were performed according to the Protection of Animals Act, after receiving approval from the German local authorities (MELUND, Kiel, Germany, application nos. V311-72241.121-4 and V242-30421/2016).

### TNFα-Induced Shock Model

Recombinant carrier-free murine TNFα was obtained from R&D Systems (Bio-Techne, Wiesbaden, Germany). Each female mouse received a single bolus of 1 mg murine TNFα/kg body weight in a total volume of 200 μl phosphate-buffered saline, via the tail vein. The animals were placed under permanent observation and survival was checked every 15 min.

### Ischemia-Reperfusion Injury (IRI)

Kidney IRI was induced via a midline abdominal incision and 40-min bilateral renal pedicle clamping using microaneurysm clamps (Aesculap, Inc.) as described previously (Moerke et al., 2018). Male mice were sacrificed 48 h after reperfusion, and serum urea and creatinine values were measured.

### Statistical Methods and Analyses

For all experiments, dataset differences were considered statistically significant when *p*-values were lower than 0.05, unless otherwise specified. Statistical comparisons were performed using the Mann–Whitney *U*-test with exception of the survival curves which were analyzed using the Gehan-Breslow-Wilcoxon test and the log rank test (Mantel-Cox). Asterisks in the figures/legends specify statistical significance (^∗^*p* < 0.05, ^∗∗^*p* < 0.02, and ^∗∗∗^*p* < 0.001). Statistics are indicated as SD, unless otherwise specified.

## Results

**FIGURE 1:**
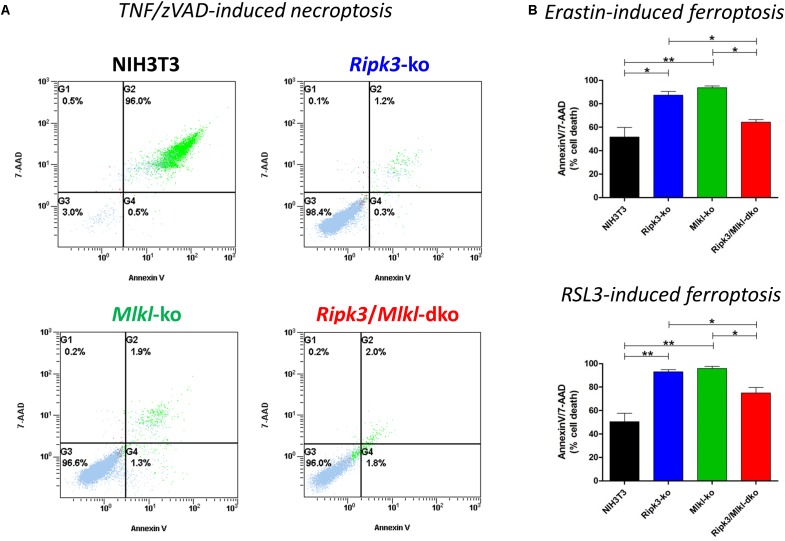
In contrast to the parental NIH3T3 cells, *Ripk*3 knockout (ko) NIH3T3 cells, *Mlkl* knockout (ko) and *Ripk3/Mlkl* double-knockout (dko) NIH3T3 cells are protected from necroptosis. **(A)** Cell death was induced by the addition of 100 ng/ml TNFα + 25 μM zVAD for 24 h at 37°C. Necroptotic cell death was quantified by FACS using 7-AAD and phosphatidylserine accessibility (annexin V staining) as markers. A representative FACS dot plot of three independent experiments is shown. **(B)** Detection of ferroptosis in NIH3T3 clones. All cells were treated for 24 h at 37°C with 2 μM erastin or 2.5 μM RSL3. Ferroptotic cell death was quantified by FACS using 7-ADD and annexin V. Results are the mean ± SD of three independent experiments. ^∗^*p* < 0.05 and ^∗∗^*p* < 0.02.

Recently, we have shown that two forms of regulated cell death, necroptosis and ferroptosis, are alternative, in that resistance to one pathway sensitizes cells to death via the other pathway, suggesting a mechanism by which one regulated pathway compensates for the other, when one is compromised (Müller et al., 2017). Regarding increased susceptibility to ferroptosis, we obtained these novel insights by specific deletion of *Mlkl*. In contrast to RIPK3, MLKL is so far known to merely play a role in necroptosis (Alvarez-Diaz et al., 2016). Here, however, we confirmed *in vitro* the aforementioned hypersensitization to ferroptosis and consequently coordinated the regulation of these two pathways in a similar manner by deleting *Ripk3* instead of *Mlkl*. However, as RIPK3 exerts its functions independently of necroptosis (Moriwaki and Chan, 2017), we were interested in examining whether the combined loss of *Ripk3* and *Mlkl* genes was equivalent to single gene knockouts. As illustrated in Figure 1A, the *Ripk3/Mlkl* dko protected the cells from TNF/zVAD-induced necroptosis just as effectively as each single knockout. As expected, our previously described time- and concentration-dependent hypersensitivity to ferroptosis in *Mlkl* knockout cells, was also present in the *Ripk3* knockout cells. To prove this, we depicted a representative experiment in which cells were treated for 24 h at 37°C with 2 μM erastin and with 2.5 μM RSL3 (Figure 1B), small molecules that trigger this unique iron-dependent form of regulated cell death (Dixon et al., 2012). However, even more astonishing were the findings regarding the *Ripk3/Mlkl* dko cells in this setting. Paradoxically, hypersensitivity of the dko cells, to erastin- and RSL3-induced ferroptosis, which was detectable across a range of erastin and RSL3 concentrations (1–10 μM, data not shown) was, in contrast to each single knockout, almost completely abrogated (Figure 1B). Although the dko cells were still protected completely from TNF/zVAD-induced necroptosis (Figure 1A), in contrast to each single knockout, the dko conferred no increased sensitization to ferroptosis and the cells behaved more like the parental unedited NIH3T3 cells. Initially, we thought that this was probably a cell-specific phenomenon, especially as the observed effect of the absent hypersensitivity of the dko cells to ferroptosis could also not be reproduced using CRISPR/Cas9-edited *Ripk1/Mlkl* double-deficient NIH3T3 cells (data not shown). However, the strong contrast of the dko of the immediately adjacent necrosome members RIPK3 and MLKL perplexed us.

**FIGURE 2:**
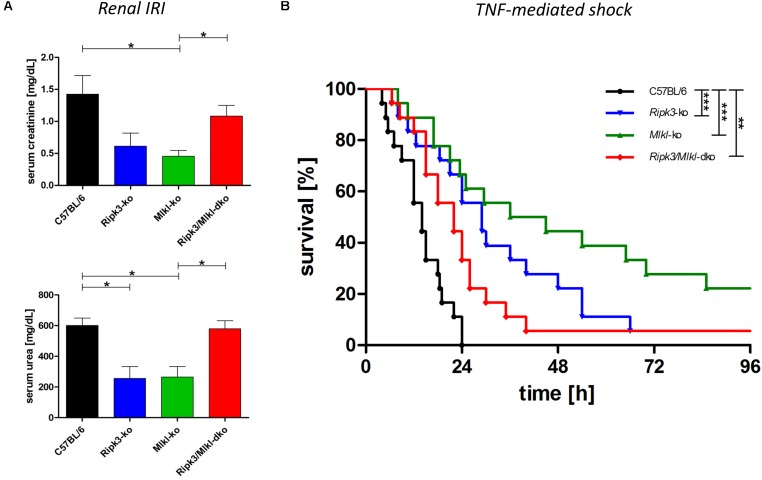
Combined knockout of *Ripk3* and *Mlkl* in an entire organism antagonizes the beneficial effect of each single knockout in ongoing necroptotic cell death processes. **(A)** For establishing renal IRI, mice (*n* = 16 per group) underwent 40-min bilateral renal pedicle clamping followed by 48-h reperfusion. The wild-type mice had notably higher plasma levels of serum creatinine and urea than the *Ripk3* and *Mlkl* knockout animals. The remarkable protection of each single knockout in the model could not be detected in the *Ripk3/Mlkl* dko animals. **(B)** *Ripk3* and *Mlkl* deficiency protects against TNF-mediated shock, induced by a high-dose TNF (1 mg murine TNFα/kg body weight). Mice with combined knockout of both genes resembled the vulnerable wild-type mice and the superior effect and survival benefit of each single knockout was abolished nearly completely in the dko model (*n* = 18 per group). Survival is presented as a Kaplan–Meier plot. ^∗^*p* < 0.05, ^∗∗^*p* < 0.02, and ^∗∗∗^*p* < 0.001.

Mouse models have been the key biological tools for defining regulated cell death in development, physiology, and homeostasis. So far, the major phenotypes observed in mice deficient in cell death pathway genes, by the intercrossing of different null alleles, have been described (reviewed in Belizario et al., 2015). Crossing transgenic knockout mouse models deficient in two and more genes has helped elucidate cell death pathways and the essential role of the downstream regulator genes involved in several inflammatory pathologies. However, in contrast to *Ripk1/Mlkl* dko animals, *Ripk3/Mlkl* dko animals are anatomically normal, viable, and fertile (Tanzer et al., 2015). For this reason, we tested *Ripk3/Mlkl* dko animals in direct comparison with both single knockouts in an acute kidney IRI model and in TNF-mediated shock. To track the benefit of genetic deficiency in core necroptotic signaling pathway components, we induced a severe renal IRI in which mice underwent 40-min of bilateral renal pedicle clamping followed by 48-h reperfusion. As shown in Figure 2A, the wild-type mice exhibited elevated serum creatinine and urea levels, indicating compromised kidney function. As described previously, *Ripk3* (Linkermann et al., 2013) and *Mlkl* knockout (Müller et al., 2017) conferred distinct protection in this model. As mentioned before, the extent of kidney damage and protection in this model depends greatly on the duration of ischemia and following reperfusion. Nevertheless, the *Ripk3/Mlkl* dko animals were, astonishingly, in contrast to the single knockout animals, barely protected in this cell death modality, but were rather comparable to their genetically unedited wild-type counterparts. Interestingly, the TNFα-mediated inflammatory *in vivo* model presented a similar scenario. *Ripk3* and *Mlkl* single deficiency each protected against TNF-mediated shock convincingly (Figure 2B), whereas dko did not. However, as we have reported previously, *Ripk3* knockout mice exhibit prolonged survival following high-dose TNFα injection (Linkermann et al., 2013). In contrast to the missing protection in this pathophysiological model of TNF-induced shock, when *Mlkl*-deficient mice received intravenous low-dose TNF (Newton et al., 2016a), their substantially prolonged survival in this setting (high-dose TNFα) resembled that of the *Ripk3*-deficient mice. However, the combined *Ripk3/Mlkl* dko nearly completely abolished the superior effect and survival benefit of each single knockout (Figure 2B), indicating that the complex regulation and interconnectivity among a single regulated necrosis pathway is still not fully understood.

## Discussion

Necroptotic cell lysis and the resultant release of proinflammatory mediators are thought to cause inflammation in necroptotic disease models. Preferentially, investigators have utilized mice lacking *Ripk3* as a necroptosis deficiency model, even where caspase inhibitors were absent under the conditions investigated. However, it is now emerging that RIPK3 almost certainly has pro-inflammatory effects clearly separable from its role in necroptosis (Kearney and Martin, 2017). Therefore, pseudokinase MLKL is currently viewed as the sole and main effector of necroptosis (Hildebrand et al., 2014). Nevertheless, it was shown recently that *Staphylococcus aureus* infection in *Ripk3-* versus *Mlkl*-deficient animals has opposing outcomes: *Mlkl* deficiency led to a delayed clearance of the bacterium, increased inflammation, and a worse outcome, whereas *Ripk3* deficiency led to an improved bacterial clearance and reduced inflammation (Kitur et al., 2016), showing that the loss of *Ripk3* is not equivalent to the loss of *Mlkl* and/or necroptosis and verifying that signaling is usually far more complex. Interestingly, to date, virtually no *in vivo* data have been published using *Ripk3/Mlkl* double-deficient animals.

Our investigation of *Ripk3* and *Mlkl* single knockout mice, in established preclinical models of severe AKI and high-dose TNF-induced shock, respectively, did not yield contrasting data. In our experience, knocking out *Ripk3* or *Mlkl* leads to remarkable and quantitatively indistinguishable protection from injury in both *in vivo* models. However, reported differences of these single-null animals are often evident in rather mild conditions triggering the abovementioned clinical disorders (Newton et al., 2016a). Nevertheless, our results regarding the *Ripk3/Mlkl* dko mice remain a conundrum. We predicted that the dko animals would receive the same protective effect in the AKI and TNF-induced shock models, as each single knockout, or rather that in their limited ability to stimulate inflammasomes and the inability to activate necroptosis, the dko mice would have increased protection against these pathologies. However, none of this occurred. The dko mice in each case unexpectedly resembled the vulnerable wild-type mice, and the former protective effect in both *in vivo* models, which was mediated by *Ripk3* or *Mlkl* loss, respectively, was completely abrogated. As all mice were backcrossed to an identical C57BL/6 background and as all animals were obtained from our facility, we postulate that differences in colony microflora, or similar, were not responsible for this discovery. Mechanistically, we eschew the hypothesis that immunoreactivity may explain our controversial findings, particularly when in a published animal model of TNF hypersensitivity, the skin principally underwent apoptosis while the spleen and liver in these identical mice were sensitized to necroptosis (Rickard et al., 2014a). So far, the physiological stimulus or insult that dictates the regulated death modality induced *in vivo* remains unmanageable, but a feasible possibility is that merely effector abundance might dictate which death signaling occurs. Nevertheless, we assessed pooled data from several independent experiments to increase the statistical power. The AKI approach was repeated twice with eight mice per group; the TNF-induced shock model was replicated three times with six animals per group. As we obtained identical results in each case, we are convinced that it is a reliable consequence of the simultaneous *Ripk3* and *Mlkl* knockout. Our data unambiguously demonstrates that necroptosis is not the only cell death modality implicated in both abovementioned pathologies and raises the question about the existing functions of RIPK3, and above all, of MLKL beyond necroptosis, and if so, how are they differentially regulated? The concept that combined deficiency in pro-inflammatory and necroptotic signaling in these models switches the etiopathology at onset completely toward ferroptosis, which we have proved, although only in the AKI model with necroptotic-resistant *Mlkl* knockout mice (Müller et al., 2017), can be verified only after developing durable, less serum-labile ferroptosis inhibitors. Furthermore, there is justifiable doubt that necroptosis is the sole initiator of inflammation. Interestingly, *Ripk3* knockout mice in a dextran sulfate sodium (DSS)-induced colitis model had enhanced sensitivity, suggesting that RIPK3 may also have tissue regenerative functions (Moriwaki et al., 2014). Presumably, processes such as mitochondrial ROS generation can contribute to necroptosis, but will be bypassed, activating the necroptotic pathway downstream at the RIPK3 or MLKL level. However, our own discovery of MLKL-independent necroptosis (Günther et al., 2016) and the recently published report of RIPK3-independent necroptosis (Zhang et al., 2016), and the unexpected function of ZBP1 (Z-DNA binding protein 1) in the skin (Lin et al., 2016) and thymus (Newton et al., 2016b), suggests that regulated necrosis pathways still have secrets and further related work would be both essential and highly informative.

## Author Contributions

SK and UK designed the research. CM and FB performed the experiments. CM, FB, UK, and SK analyzed the data. CM prepared the figures. SK wrote the paper.

## Conflict of Interest Statement

The authors declare that the research was conducted in the absence of any commercial or financial relationships that could be construed as a potential conflict of interest.
